# Role of hepatic PKC**β** in nutritional regulation of hepatic glycogen synthesis

**DOI:** 10.1172/jci.insight.149023

**Published:** 2021-10-08

**Authors:** Yaoling Shu, Faizule Hassan, Michael C. Ostrowski, Kamal D. Mehta

**Affiliations:** 1 Department of Biological Chemistry & Pharmacology, The Ohio State University Wexner Medical Center, Columbus, Ohio, USA.; 2 Department of Biochemistry & Molecular Biology, Holling Cancer Center, Medical University of South Carolina, Charleston, South Carolina, USA.; 3 Instacare Therapeutics, Dublin, Ohio, USA.

**Keywords:** Hepatology, Glucose metabolism

## Abstract

The signaling mechanisms by which dietary fat and cholesterol signals regulate central pathways of glucose homeostasis are not completely understood. By using a hepatocyte-specific PKCβ-deficient (PKCβ^Hep–/–^) mouse model, we demonstrated the role of hepatic PKCβ in slowing disposal of glucose overload by suppressing glycogenesis and increasing hepatic glucose output. PKCβ^Hep–/–^ mice exhibited lower plasma glucose under the fed condition, modestly improved systemic glucose tolerance and mildly suppressed gluconeogenesis, increased hepatic glycogen accumulation and synthesis due to elevated glucokinase expression and activated glycogen synthase (GS), and suppressed glucose-6-phosphatase expression compared with controls. These events were independent of hepatic AKT/GSK-3α/β signaling and were accompanied by increased HNF-4α transactivation, reduced FoxO1 protein abundance, and elevated expression of GS targeting protein phosphatase 1 regulatory subunit 3C in the PKCβ^Hep–/–^ liver compared with controls. The above data strongly imply that hepatic PKCβ deficiency causes hypoglycemia postprandially by promoting glucose phosphorylation via upregulating glucokinase and subsequently redirecting more glucose-6-phosphate to glycogen via activating GS. In summary, hepatic PKCβ has a unique and essential ability to induce a coordinated response that negatively affects glycogenesis at multiple levels under physiological postprandial conditions, thereby integrating nutritional fat intake with dysregulation of glucose homeostasis.

## Graphical abstract

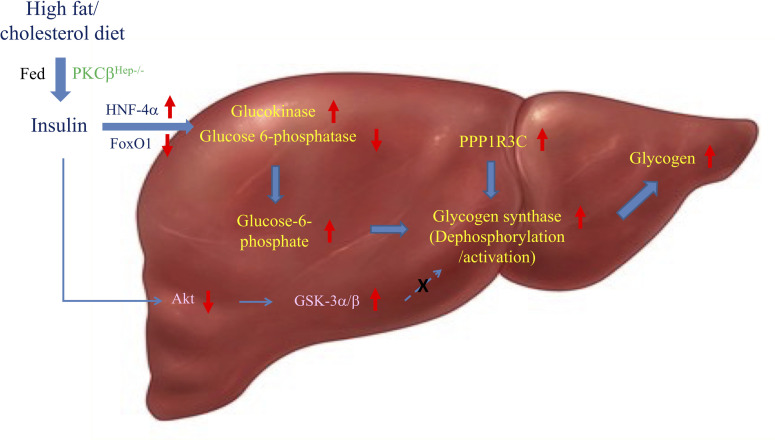

## Introduction

The liver plays an important role in maintaining plasma glucose homeostasis by adjusting a delicate balance between hepatic glucose utilization and production, derangements of which occur in metabolic diseases (1, 2). After a meal, the liver adapts to feeding through several events, including suppressing gluconeogenesis and upregulating glycogen synthesis, which result in a net switch from hepatic glucose output to hepatic glucose uptake. These adaptive responses are critical for maintaining glucose homeostasis in response to glucose overload. The adaptive mechanism is a complex process and is regulated directly and indirectly via diverse mechanisms, including transcriptional and posttranscriptional mechanisms by hormones, nutrients, and other cues. In response to ingestion of glucose and the resulting hyperinsulinemia and hyperglycemia, fasted-to-fed transition in the liver is accomplished by promoting expression of genes normally involved in glycogen synthesis. Conversely, genes that are required for gluconeogenesis are downregulated. These physiologically opposed cascades are regulated, at least in part, at the transcriptional level of the glucokinase (GK) and glucose-6-phosphatase (G6Pase) genes, which catalyze the first and last rate-limiting steps in glycogenesis and gluconeogenesis, respectively (3). GK is considered to play an essential role in sensing and maintaining proper blood glucose levels in response to a rising level of glucose. This kinase mediates hepatic glucose uptake, and its product glucose-6-phosphate (G6P) is a central metabolite for intrahepatic glucose homeostasis. This metabolite serves as the substrate for glycogen synthesis and acts as an allosteric activator of glycogen synthase (GS), the rate-limiting enzyme for glycogen synthesis (4–6). The activity of GS is normally regulated by phosphorylation and inactivation at multiple sites, but hyperphosphorylated GS can be fully activated by saturating concentrations of G6P (5). The above studies established interplay among GK, G6Pase, and GS in glucose homeostasis; however, the underlying molecular and signaling mechanisms linking these critical regulators are incompletely understood. In addition, little is known about what determines coordinated activation or repression of hepatic gene expression and activity of these genes in vivo. Well-coordinated hepatic glucose metabolism is essential to health, and dysregulation of hepatic glucose metabolism is central to the pathogenesis and complications of type 2 diabetes mellitus.

Several epidemiological studies have linked development of metabolic disruptions to altered lipid metabolism and dietary fat intake (7). Consumption of a Western diet enriched with high fat and high cholesterol is reported to aggravate glucose homeostasis disorders, but the mechanisms underpinning such an association are not fully defined (7–9). Dietary nutrients interact with gene networks to orchestrate adaptive responses during metabolic stress. Considerable progress has been made in identifying transcriptional and posttranscriptional mechanisms in response to feeding; however, molecular mechanisms by which dietary fat/cholesterol excess are integrated and sensed by glucose homeostatic machinery remain elusive. Ambiguity persists on the identity and interactions of signaling pathways by which dietary fat metabolites and signals impinge on these mechanisms to switch the liver from net intake to net output of glucose. Understanding of how dietary signals influence glucose homeostasis in various physiological situations or metabolic disorders is crucial for limiting the development of metabolic pathologies.

Protein kinase C β (PKCβ) activated by dietary fat and metabolites (diacylglycerol, saturated fatty acids, and cholesterol), glucose, and calcium has been implicated in the regulation of metabolism (10–14). Obesity is associated with higher PKCβ levels in humans, and high-fat feeding upregulates PKCβ expression in mice (14, 15). Importantly, whole-body PKCβ-deficient mice are protected against diet-induced adiposity, hepatic steatosis, and development of insulin resistance; however, the underlying mechanisms are not fully understood. To elucidate how PKCβ regulates energy homeostasis, we undertook a loss-of-function approach by deleting the coding DNA sequence for the catalytic domain of the PKCβ gene in the liver (PKCβ^Hep–/–^) and monitored susceptibility to Western diet–induced hyperglycemia under different conditions. Here, we provide genetic evidence that hepatic PKCβ induces a coordinated response at multiple levels to negatively regulate glycogen storage in the liver under physiological postprandial conditions. Hepatic PKCβ deficiency in vivo caused hypoglycemia by promoting glucose phosphorylation through upregulating GK transcription and concomitantly redirecting more G6P to glycogen via activating GS, independent of AKT/GSK-3α/β signaling. It is likely that the hepatic PKCβ level in the cell could dictate the magnitude of glycogen synthesis and deposition, thus allowing fine-tuning of the pathway based on changing cell conditions. Taken together, our data strongly imply that PKCβ has the unique and essential ability to suppress hepatic glycogen synthesis under physiological postprandial conditions, thereby contributing to dietary fat–induced dysregulation of glucose homeostasis.

## Results

### Hepatic PKCβ deficiency promotes glucose disposal under fed conditions.

To define the physiological and molecular consequences of depleted hepatic PKCβ signaling on glucose homeostasis, we generated PKCβ^Hep–/–^ mice depleted of PKCβ in hepatic cells by crossing floxed PKCβ mice (PKCβ^fl/fl^) with albumin-Cre mice (16). PKCβ deficiency was verified by specific immunoblotting and analysis of tissues revealed that the absence of PKCβ was restricted to the liver of these mice.

**Figure 1:**
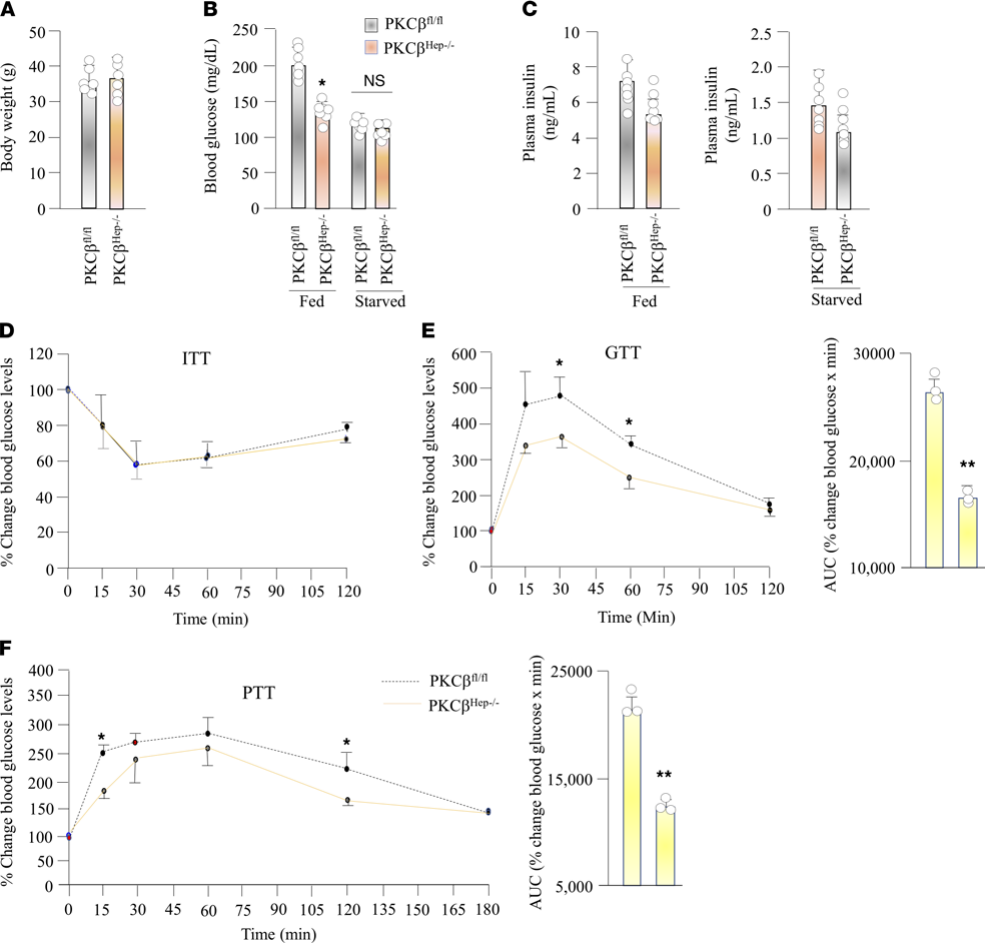
Hepatic PKCβ deficiency promotes postprandial hypoglycemia. (**A**) Comparison of body weight between PKCβ^fl/fl^ and PKCβ^Hep–/–^ mice fed HFHC diet for 12 weeks. (**B** and **C**) Fed and fasted (12–16 hours) blood glucose and serum insulin concentrations in the above mice. (**D**) ITT: control and KO mice were fasted overnight before injection of 0.5 units/kg body weight. Blood samples were collected through the tail vein and glucose concentrations were measured before and after 30, 60, 90 and 120 minutes after the challenge by glucometer. (**E**) PTT: control and KO mice were fasted overnight before i.p. injection of 2 gm of pyruvate per kg body weight. Blood glucose concentrations were measured from the tail vein at indicated time. (**F**) GTT: control and KO mice were fasted overnight before i.p. injection of 1 g glucose per kg body weight. Results were calculated as percentage of blood glucose values at time 0 for each mouse. AUC was calculated for both GTT and PTT using GraphPad software. Data are presented as the mean ± SEM of 2 independent experiments of triplicate measurements. (*n* = 3–6). *P < 0.05, **P < 0.01 for 2-tailed *t* tests (**A**–**C**) or 2-way ANOVA followed by Bonferroni’s multiple-comparison test (**D**–**F**).

To investigate the consequences of depleted hepatic PKCβ signaling on glucose homeostasis, we first monitored blood glucose levels in the high-fat and high-cholesterol diet–fed (HFHC diet–fed) control and KO mice under different conditions. PKCβ^Hep–/–^ mice had a similar body weight and fasting glucose levels; however, these mice exhibited a significant decrease in blood glucose levels by 34% in mice under the fed condition (Figure 1, A and B). Interestingly, blood insulin levels trended lower in PKCβ^Hep–/–^ mice relative to controls, but never reached a significant difference under either fasted or fed conditions (Figure 1C), suggesting that hypoglycemia in PKCβ^Hep–/–^ mice may not be caused by improvement in insulin sensitivity. Accordingly, insulin tolerance tests (ITTs) revealed no significant differences between PKCβ^Hep–/–^and control mice (Figure 1D) (16). To examine what caused blood glucose lowering in PKCβ^Hep–/–^ mice, we performed the following tests. The glucose tolerance test (GTT) also showed that PKCβ^Hep–/–^ mice had moderately improved glucose tolerance relative to control mice (Figure 1E). The pyruvate tolerance test (PTT), which measures the rate of de novo glucose synthesis, showed that inactivation of hepatic PKCβ improved tolerance to exogenous pyruvate injection in PKCβ^Hep–/–^ mice relative to controls, suggesting that hepatic gluconeogenesis may be decreased in the absence of PKCβ (Figure 1F). The AUC of the overall pyruvate tolerance and glucose tolerance was decreased by 38% and 40% in the PKCβ^Hep–/–^ mice relative to the control, respectively (Figure 1, E and F).

**Figure 2:**
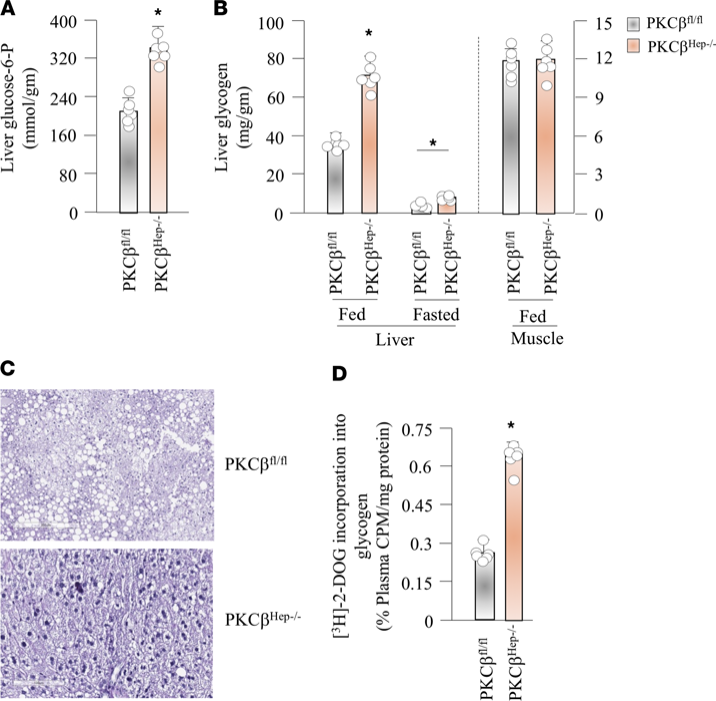
Hepatic PKCβ deficiency promotes glycogen accumulation in the liver. (**A**) Comparison of G6P content in the liver of PKCβ^fl/fl^ and PKCβ^Hep–/–^ mice fed HFHC diet for 12 weeks. (**B**) Glycogen content in liver and skeletal muscle (hind limb) under fed condition and in the liver after a 16-hour fast. (**C**) Representative PAS staining of liver from above HFHC-fed PKCβ^fl/fl^ and PKCβ^Hep–/–^ mice. (**D**) 2-Deoxy-D-[^3^H] glucose incorporation into glycogen measured after 12–16 hours of fasting in PKCβ^fl/fl^ and PKCβ^Hep–/–^ mice fed HFHC diet for 12 weeks. Label incorporation was measured as cpm from precipitated glycogen from liver tissue lysate and normalized to plasma cpm/mg protein. The results are mean ± SEM. *n* = 3–6 mice/genotype in each group. *P < 0.05 versus control mice by 2-tailed *t* tests (**A** and **D**) or 2-way ANOVA followed by Bonferroni’s multiple-comparison test (**B**). Total original magnification, ×200.

Liver glycogen plays a crucial role in maintaining blood glucose homeostasis. We therefore compared hepatic G6P and glycogen contents under fed and fasted conditions. Unlike mice fed a chow diet, hepatic G6P and glycogen content were significantly increased in the ad libitum fed state in PKCβ^Hep–/–^ mice fed an HFHC diet compared with control mice (Figure 2, A and B, and Supplemental Figure 1; supplemental material available online with this article; https://doi.org/10.1172/jci.insight.149023DS1). After an overnight fast, PKCβ^Hep–/–^ mice showed decreased hepatic glycogen content compared with the fed state. This observation indicates net mobilization of liver glycogen, although this glycogen was not depleted to the same extent as in control mice (Figure 2B). Moreover, skeletal muscle glycogen content was similar between the groups, and thus consistent with the idea that glycogen synthesis in nonhepatic tissues was not altered (Figure 2B). Accordingly, periodic acid-Schiff staining of liver sections showed greater positive staining for glycogen in the PKCβ^Hep–/–^ liver as indicated by more purple color compared with control livers (Figure 2C). The increase in glycogen was not sufficient to compensate for reduction in lipid content of the PKCβ^Hep–/–^ liver because glycogen-associated water content only accounts for 5% of liver mass (16). The weight of the liver of HFHC-fed PKCβ^Hep–/–^ mice was lower relative to the control liver (16).

Stimulation of liver glycogen synthesis is a major direct effect of insulin on the hepatocyte and its elevation results in postprandial hypoglycemia. To compare the glycogen synthesis rate in livers, we performed 2-deoxy-D-[^3^H] glucose incorporation into glycogen as a surrogate measurement of GS activity. Radioactivity in the glycogen pellet from the liver was measured at 60 minutes and quantified as a percentage of glucose-specific activity of total radioactivity in plasma and of total protein content. In this experiment, PKCβ^Hep–/–^ mice exhibited significantly elevated GS activity in the liver (Figure 2D). Thus, the loss of hepatic PKCβ resulted in increased GS activity and glycogen content in the ad libitum fed state.

### Hepatic PKCβ deficiency alters expression of metabolic genes promoting glycogenesis and reducing gluconeogenesis.

**Figure 3:**
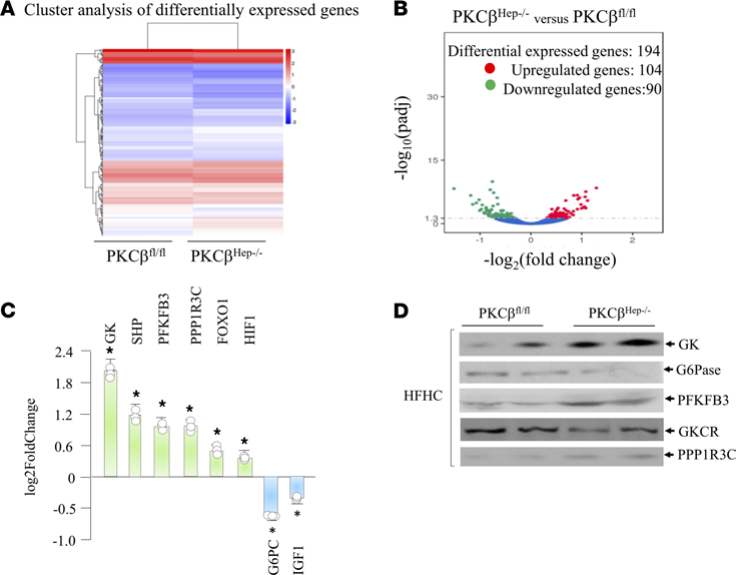
Hepatic PKCβ deficiency alters expression of genes promoting glycogenesis and gluconeogenesis. RNA-Seq analysis of liver mRNA from PKCβ^fl/fl^ and PKCβ^Hep–/–^ mice fed high-fat diet (HFD) for 12 weeks. (**A**) Hierarchical clustering heatmap of differentially expressed genes in the liver of PKCβ^fl/fl^ and PKCβ^Hep–/–^ mice fed HFD for 16 weeks. Red represents high expression genes; blue represents low expression genes. Color descending from red to blue indicates log_10_(FPKM + 1) from large to small. (**B**) KEGG enrichment histogram analysis. (**C**) Genes related to glycogen metabolism are differentially expressed in liver of the above mice. (**D**) Western blotting of liver extracts from above mice for GK, G6Pase, PFKFB3, and GKCR proteins. The results are mean ± SEM. *P < 0.05 indicates the difference between control and KO livers. (*n* = 3) by 2-way ANOVA followed by Bonferroni’s multiple-comparison test (**C**).

Regulation of hepatic gene expression is largely responsible for the control of glucose metabolism. To assess molecular changes that lead to the phenotypic differences between genotypes, we performed RNA-Seq to determine differential expression of genes involved in glucose metabolism between control and PKCβ^Hep–/–^ livers. RNA-Seq analysis on liver RNA identified genes selectively altered in the liver of PKCβ^Hep–/–^ mice relative to controls (Figure 3A). There were 194 genes differentially expressed; 104 genes were upregulated and 90 genes were downregulated between genotypes (Figure 3B). Altered expression of selected genes involved in glycogen metabolism can be linked to a significantly higher liver glycogen level in PKCβ^Hep–/–^ mice compared with controls (Figure 3C). We found that genes that encode enzymes critical in the determining G6P levels were upregulated in the PKCβ^Hep–/–^ liver compared with the control liver. These included GK and phosphofructokinase-2/fructose bisphosphatase-2 (PFK2/FBP2), whereas expression of G6Pase, the final gatekeeper for glucose efflux from the cell that catalyzes the last step of gluconeogenesis, exhibited downregulation. We also observed upregulation of protein phosphatase 1 bound to glycogen targeting subunit 3C (PPP1R3C; also known as PTG), which removes the phosphate group from GS. In addition, small heterodimer partner (SHP) exhibited significantly increased expression in the PKCβ^Hep–/–^ liver compared with the control liver. Expression of hepatocyte nuclear factor 4α (HNF-4α) did not show any difference, whereas expression of FoxO1 was slightly upregulated. Since these genes are directly or indirectly involved in glycogen synthesis, these results suggest that hepatic PKCβ regulates glycogen content by controlling expression of genes involved in glycogen synthesis. Lowering of G6Pase expression may also account for mildly reduced gluconeogenesis (Figure 1F). The above results were verified using quantitative PCR (qPCR) on liver mRNA from both genotypes. Finally, Western blot analyses also confirmed increased expression of GK, PPP1R3C, and PFKFB3 and decreased expression of G6Pase (Figure 3D). We also found reduced expression of GK regulatory protein (GKCR) in the PKCβ^Hep–/–^ liver compared with the control (Figure 3D). This protein regulates a nuclear-cytosolic translocation cycle that determines GK activity. Collectively, these data suggest that PKCβ deficiency caused significant perturbation in nutrient balance that favored shunting of glucose into glycogenic pathway in the fed state.

### PKCβ deficiency alters the insulin signaling pathway to promote glycogenesis postprandially.

**Figure 4:**
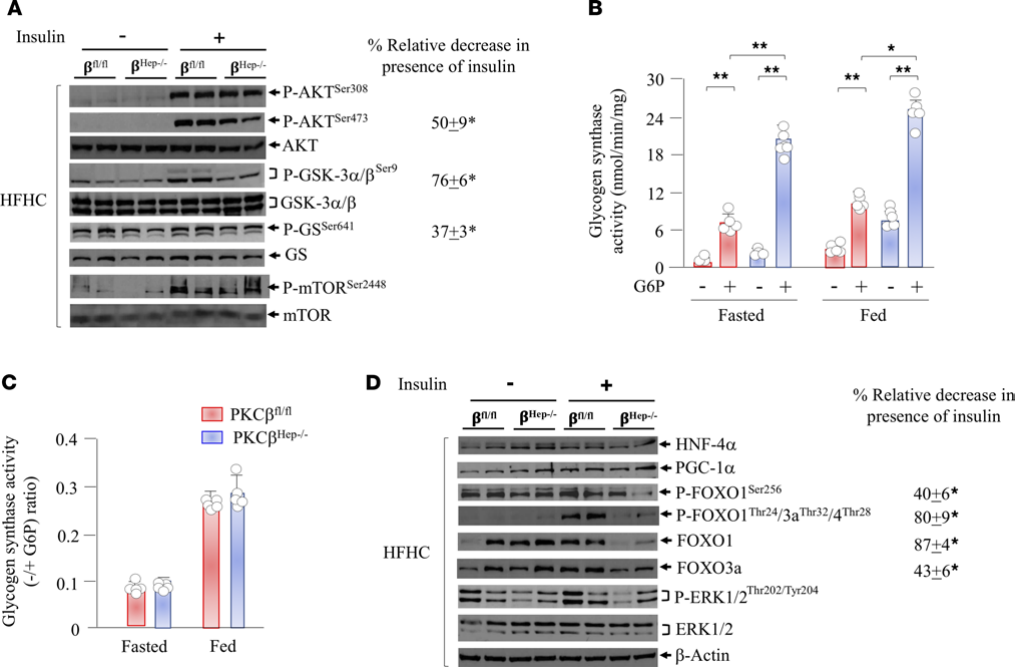
Hepatic PKCβ deficiency alters insulin signaling to promote FoxO1 degradation and keep GS activated postprandially. (**A** and **D**) Immunoblot analyses of indicated phospho- and total proteins in the liver of HFHC-fed PKCβ^fl/fl^ and PKCβ^Hep–/–^ mice injected either with saline or insulin (5 U/kg body weight). Liver was harvested after 12 minutes, and liver extracts were subjected to SDS-PAGE and immunoblotted with indicated antibodies. Western blots are representative of 2 separate experiments performed with tissue from 4 mice. Percentage relative decrease shows the band intensity ratio of PKCβ^Hep–/–^ over PKCβ^fl/fl^. (**B** and **C**) GS activity was measured in liver lysates + 10 mmol/L G6P and are presented as total GS activity (**B**) or the –/+G6P ratio (**C**). Each reaction was performed in triplicate. Each value represents the mean ± SEM. *n* = 4–6 mice/genotype; *P < 0.05 and **P < 0.005 for 2-way ANOVA followed by Bonferroni’s multiple-comparison test.

Since insulin signaling affects glycogen synthesis, we compared insulin signaling pathways in the livers of control and PKCβ^Hep–/–^ mice. To this end, we injected mice fasted 6 hours with an i.p. bolus of insulin and sampled livers 12 minutes later to assess the phosphorylation status of proteins critical for glycogen synthesis, namely glycogen synthase kinase-3α/β (GSK-3α/β) and GS, by using phosphorylation state–specific antibodies. As expected, control PKCβ^fl/fl^ mice injected with a bolus of insulin demonstrated a marked increase in phosphorylation of liver GSK-3α/β compared with the PKCβ^Hep–/–^ liver (Figure 4A; full uncut gels are available in the supplemental material). Densitometric analysis of phospho- and unphosphorylated GSK-3α/β revealed that phosphorylation of GSK-3α/β (Ser21/9) was significantly lower (>3-fold) in the liver of PKCβ^Hep–/–^ mice compared with controls. Because phosphorylation of GSK-3α/β is a key step for its inactivation by insulin, we examined phosphorylation/inactivation status of the downstream substrate GS. GSK-3α/β predominantly phosphorylates GS at different sites, including critical residue GS-Ser640/641. The basal level of phosphorylation of downstream GS-Ser641, normalized to total GS protein, was lower in the PKCβ^Hep–/–^ liver compared with the PKCβ^fl/fl^ liver. In response to insulin, no reduction in GS phosphorylation in the HFHC-fed mouse PKCβ^fl/fl^ liver points to impaired insulin signaling to GS caused by high-fat diet. In line with this possibility, insulin caused a significant reduction in GS phosphorylation in the chow-fed mouse PKCβ^fl/fl^ liver (Supplemental Figure 2). Notably, the PKCβ^Hep–/–^ liver exhibited significant reduction in GS-Ser641 phosphorylation both in the absence and presence of insulin, without any effect on protein levels of GS. The ratio of GS-Ser641 phosphorylated to total GS, which determines the activity status of GS, was decreased, suggesting that GS was less phosphorylated in the PKCβ^Hep–/–^ liver compared with the PKCβ^fl/fl^ liver (Figure 4A). In accordance, PKCβ^Hep–/–^ liver lysates exhibited significantly higher GS activity without affecting G6P-dependent GS activation under both fasted and fed conditions (Figure 4, B and C). It is likely that increased glycogen synthesis in the PKCβ^Hep–/–^ liver is mainly due to a significant increase in G6P levels, leading to activation of less phosphorylated GS.

Protein kinase B (AKT) has been reported to phosphorylate both isoforms of GSK-3 on their N-terminus; in the case of GSK-3α, position S21 is targeted, whereas GSK-3β is phosphorylated at position S9. The phosphorylation of the N-terminus results in the inactivation of both GSK-3 isoforms by 40%–50%. We therefore evaluated phosphorylation status of AKT in the above livers and found that insulin-induced AKT-T308 phosphorylation was indistinguishable between control and PKCβ^Hep–/–^ livers, whereas Akt-S473 phosphorylation was reduced in the PKCβ^Hep–/–^ liver compared with the control liver, suggesting that PKCβ might even contribute to the phosphorylation of AKT (Figure 4A) (16). Indeed, PKCβ has been reported to phosphorylate AKT-S473 in a cell- and stimulus-specific manner (17). According to a recent study (18), phosphorylation at Thr-308 is necessary and sufficient for maximal AKT activity, whereas Ser-473 phosphorylation may function to tune rather than activate AKT. Previous work also found that Ser-473 was not essential for phosphorylation of GSK-3α/β (18). Interestingly, phosphorylation of another AKT substrate, mTOR-Ser2449, was comparable between genotypes (16). These results argue that a distinct kinase can phosphorylate GSK-3, with or without AKT, for the control of its activity in the liver. Interestingly, in addition to AKT, other kinases have been reported to phosphorylate and inactivate GSK-3α/β, including PKC (19–22). We performed an in vitro kinase assay in which indicated amounts of recombinant active GSK-3β protein and active recombinant PKCβ were incubated in a protein kinase assay buffer system and then resolved by SDS-PAGE followed by immunoblotting using phospho-GSK-3 antibody. We showed that PKCβ directly phosphorylated GSK-3/β in a dose-dependent manner (Supplemental Figure 3).

Insulin also induces glycogen synthesis via stimulating AKT-dependent inhibitory phosphorylation of FoxO1, which is thought to be the master regulator of key gluconeogenic genes, leading to the subsequent regulation of glucose output (23, 24). In support of this notion, ablating hepatic FoxO1 in insulin-resistant models improves glucose homeostasis (25). AKT has been shown to phosphorylate FoxO1 on Ser253, which primes phosphorylation of the other 2 sites at Thr24 and Ser319, thereby enhancing FoxO1 interaction with E3 ubiquitin ligase, and promotes FoxO1 nuclear export and/or degradation (23). To test whether hepatic PKCβ deficiency alters phosphorylation of FoxO1, we used an antibody that recognizes FoxO1 that is phosphorylated on Ser253. We found that FoxO1 was phosphorylated on this site even in the unstimulated liver, whereas FoxO1 phosphorylation and endogenous levels were dramatically reduced in the insulin-treated PKCβ^Hep–/–^ liver compared with the control liver (Figure 4D). We also found reduced endogenous FoxO3 levels in the insulin-treated PKCβ^Hep–/–^ liver compared with the control. To our surprise, FoxO1 levels changed even in the absence of any observable changes in AKT activity. Both FoxO1 and 3 have been shown to be phosphorylated by ERK at multiple residues, which can lead to both nuclear exclusion and degradation (26, 27). We therefore compared P-ERK and total ERK status and found reduced P-ERK levels in the PKCβ^Hep–/–^ liver compared with the control, suggesting that ERK is not contributing to reduction of either the FoxO1 or FoxO3 level. On the other hand, total protein levels of liver HNF-4α and PGC-1α were similar between genotypes (Figure 4D). Also, β-actin protein levels were similar in all lanes, indicating symmetrical loading of the gel. It is interesting to note that reduction in endogenous FoxO1 protein level by insulin in the PKCβ^Hep–/–^ liver was observed, whereas FoxO1 mRNA levels exhibited a slight increase, suggesting a compensatory transcriptional mechanism. We conclude that the increase in liver glycogen content correlated with changes in the insulin signaling pathway of the PKCβ^Hep–/–^ liver compared with controls.

### PKCβ deficiency promotes hepatic GK expression through transactivating HNF-4α and reducing endogenous FoxO1 occupancy.

**Figure 5:**
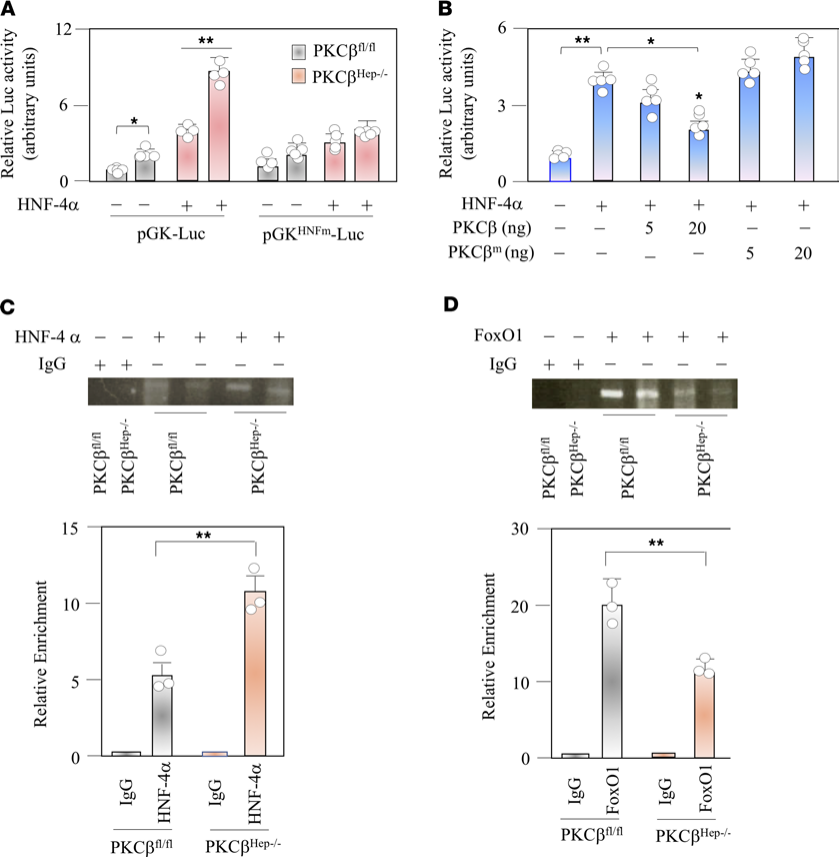
Hepatic PKCβ deficiency transactivates HNF-4α and promotes its occupancy while reducing FoxO1 occupancy at the GK promoter. (**A**) PKCβ^fl/fl^ and PKCβ^Hep–/–^ hepatocytes were transiently cotransfected with either WT pGK-Luc (300 ng) or HNF-4α site mutated reporter plasmid (pGK^HNFm^-Luc) construct (300 ng) with or without expression vector for human HNF-4α (50 ng). After transfection, cells were incubated in DMEM supplemented with 10% FBS for 12 hours and then serum-starved for 9 hours in the presence or absence of 100 nM insulin. (**B**) HepG2 cells were cotransfected with pGK-Luc (100 ng) along with expression vector for human HNF-4 (20 ng) with or without human PKCβ expression. Transfected cells were treated with insulin as described above. In each experiment, luciferase activity was presented as fold change with respect to transfection of pGK-Luc reporter plasmid alone. The values represent mean ± SEM of 3 independent experiments. (**C** and **D**) Binding of FoxO1 and HNF-4α were analyzed by ChIP assays with chromatin isolated from mice liver in the fed state. As a control, chromatin was immunoprecipitated with nonspecific IgG. Bound DNA was analyzed by PCR and images were quantified. Enrichment compared with input DNA was calculated. Results were normalized to precipitation using IgG in the fed state. Values are mean ± SEM for triplicates. *P < 0.05 for 2-way ANOVA followed by Bonferroni’s multiple-comparison test. **P < 0.01.

Among the genes with an altered expression in the PKCβ^Hep–/–^ liver, we focused on induction of GK expression because of its critical role in hepatic glucose metabolism. GK expression in the liver is dynamically regulated upon nutritional conditions. Interactions between HNF-4α and FoxO1 contribute to the differential regulation of GK and G6Pase genes by insulin (28, 29). Unlike FoxO1, the hepatic HNF-4α protein level did not change with PKCβ deficiency (Figure 4D). We first investigated the effect of PKCβ deficiency on HNF-4α transactivation potential by using transfection assays. We carried out luciferase assays using a GK promoter–driven luciferase construct and HNF-4α construct transfected in either WT or PKCβ KO hepatocytes. As shown in Figure 5A, PKCβ deficiency significantly induced GK promoter activity in hepatocytes. To further assess the effects of PKCβ on HNF-4α–mediated transcription, we transfected into HepG2 cells either a WT or catalytically inactive mutant of PKCβ together with a GK promoter–driven luciferase construct and HNF-4α. As expected, overexpression of PKCβ suppressed HNF-4α activity, whereas overexpression of the kinase-defective mutant of PKCβ had no significant effect (Figure 5B). These findings showed that PKCβ negatively regulated GK promoter activity, possibly involving HNF-4α.

To examine whether PKCβ affects recruitment of HNF-4α and/or FoxO1 to the GK promoter, we examined the effects of PKCβ deficiency on the recruitment of HNF-4α to the HNF-4α binding site, which was previously identified at –52/–39 bp upstream from the transcription initiation site in the GK promoter. As shown in Figure 5C, ChIP studies with an antibody against HNF-4α and primers that amplify the region between –219 and +52 bp confirmed that HNF-4α interacted with this part of the GK promoter and occupancy was higher in the PKCβ^Hep–/–^ liver. In contrast, PCR after IP with antibodies against FoxO1 showed that hepatic PKCβ deficiency markedly suppressed recruitment of FoxO1 to this region of the GK promoter (Figure 5D). These results indicate that PKCβ promoted and suppressed recruitment of HNF-4α and FoxO1 to its binding site in the proximal GK promoter, respectively.

## Discussion

The centrality of the liver in the maintenance of whole-body glucose homeostasis is well established (1). Glycogen metabolism is a critical mediator of this function because liver glycogen plays a crucial role in maintaining blood glucose homeostasis; disruption results in postprandial hyperglycemia (30). The importance of glycogen metabolism is highlighted by human genetic disorders that are caused by mutations in the enzymes involved. The control of glycogen synthesis involves multiple insulin-regulated enzymes, and these activities coordinately change with insulin by an incompletely understood mechanism. In the present study, by using a PKCβ^Hep–/–^ mouse model, we showed that hepatic PKCβ deficiency induced a coordinated response to synchronously induce GK transcription and suppress G6Pase expression and at the same time increase GS activity. As a result, PKCβ deficiency contributed to disposal of glucose loads by increasing the rate of glycogen synthesis while modestly suppressing gluconeogenesis; this resulted in a net switch from hepatic glucose output to hepatic glucose uptake under fed conditions. The balance between the activating and repressive functions of PKCβ may be crucial in the liver because, by repressing GK and by inducing G6Pase, PKCβ regulates the GK/G6Pase, intracellular G6P levels, and hepatic glucose handling.

### PKCβ regulates GSK-3α/β phosphorylation and suppress glycogen synthesis independent of AKT/GSK-3α/β signaling axis.

The insulin/AKT/GSK3 signaling axis is believed to be the most important pathway that regulates glycogen synthesis and is responsible for glucose clearance (3). The mechanisms regulating GSK-3α/β have proven to be a topic of great debate because they are varied and not fully understood. We showed here for the first time, to our knowledge, that insulin-induced GSK-3 phosphorylation in vivo requires PKCβ. Figure 4A shows that PKCβ deficiency caused a significant decrease in insulin-induced GSK-3 phosphorylation, even though it activated AKT to the same extent. This indicates that although AKT activation may be sufficient to inhibit GSK-3α/β activity in some cell types (31), insulin-induced full phosphorylation and inhibition of GSK-3α/β in the liver is mediated by a kinase other than AKT. Interestingly, several PKC isoforms can phosphorylate GSK-3α/β in vitro (20–22). Moreover, treating cells with phorbol esters, compounds that activate both classical and novel PKC isoforms, can inhibit GSK-3 activity (22). These results provide evidence that PKCβ is involved in GSK-3α/β phosphorylation and can link insulin and the GSK-3α/β pathway. The results argue against the sole role of AKT in the regulation of GSK-3α/β phosphorylation in the liver.

Our results support the prevailing view that an AKT/GSK-3–independent pathway mediates insulin-stimulated liver glycogen synthesis and deposition in the postprandial state (32–34). They also provide insights into the role of PKCβ in both acute and chronic regulation of hepatic GS activity under fed conditions. It is likely that elevated intracellular G6P levels due to strong induction of GK activity by hepatic PKCβ deficiency allosterically stimulates GS, which drives G6P into glycogen as a feed-forward mechanism. In addition, this effect could be further enhanced in the PKCβ^Hep–/–^ liver when GS is dephosphorylated by induction of PPP1R3C because this can make GS more sensitive to G6P and can further potentiate activity (35). Accordingly, PPP1R3C encodes a carbohydrate binding protein that is a subunit of the protein phosphatase 1 (PP1) complex, which affects glycogen biosynthesis by activating GS and limiting glycogen breakdown by reducing glycogen phosphorylase activity. Binding of G6P is known to induce a conformational shift that promotes recruitment of PPP1R3C that dephosphorylates and activates GS (4). In agreement, hepatic PPP1R3B transgenic mice show increased hepatic glycogen and improved glucose sensitivity (35). Furthermore, PKC has been shown to activate PP1 activity, and PP1 has been linked to inhibition of PKC activity (36, 37). Moreover, an increase in PP1 activity can also inhibit glycogenesis via dephosphorylation/inactivation of glycogen phosphorylase. Thus, the PKCβ/PP1 axis can provide an exquisite mechanism for limiting the action of GSK3 on GS phosphorylation/inactivation in the liver. It is important to note in this regard that hepatic GS is shown to be phosphorylated by Ca^2+^-sensitive PKC, but PKC-induced phosphorylation did not cause inactivation, thus ruling out a direct role of PKCβ in controlling GS activity (38).

It can be concluded that PKCβ deficiency has a composite effect in controlling glycogen synthesis through allosteric activation and efficient dephosphorylation of GS, thus both producing and redirecting more G6P derived from blood glucose to glycogen. Importantly, GK has a close functional and regulatory association with glycogen synthesis, and GK and GS enzymes are reported to be located in the same peripheral areas of hepatocyte cytoplasm in which glycogen synthesis occurs (38). Accordingly, concomitant overexpression of both GK and GS in mouse models enhanced hepatocellular glycogen synthesis more than the overexpression of either gene alone (6). Previous studies have also shown that diabetic patients have lower hepatic glycogen content due to decreased GS and/or increased glycogen phosphorylase activity (39, 40). This can result in a shift of G6P flux into glycolytic and lipogenic pathways, leading to hyperlactemia and hyperlipidemia, respectively. The ability of the PKCβ^Hep–/–^ liver to redirect more of G6P derived from gluconeogenesis and blood glucose to glycogen can effectively suppress de novo lipid synthesis, in spite of elevated hepatic GK expression/activity. In addition, we recently reported that PKCβ positively regulates SREBP-1c activity, and reduction of its activity in the PKCβ^Hep–/–^ liver leads to decreased expression of lipogenic genes, which could further contribute to hypolipidemia observed in these mice (16). In view of the above discussion, it is tempting to speculate that hepatic diet-induced PKCβ induction may impair the ability of the liver to synthesize glycogen and promote lipogenesis in the diabetic state.

### PKCβ deficiency potentiates differential regulation of GK and GS genes through promoting HNF-4α transactivation and reducing FoxO1 protein abundance.

Our results extend our understanding of the mechanisms mediating differential regulation of GK and G6Pase genes in vivo. Hepatic PKCβ deficiency potentiates GK induction while further suppressing G6Pase expression in the liver, which can enhance the conversion of glucose to glycogen and lower hepatic glucose production, thereby reducing postprandial blood glucose levels. We propose that hepatic PKCβ deficiency also modulates the functional interplay between HNF-4α and FoxO1 by promoting HNF-4α transactivation and sensitizing insulin-induced FoxO1 degradation. It is consistent with a previous report showing that hepatocyte-specific HNF-4α deletion resulted in depletion of hepatic glycogen during fed conditions (41). Although the present study focused on the role that HNF-4α plays in mediating the effects of insulin on GK expression, it is likely that HNF-4α and its interaction with FoxO1 may also contribute to the suppression of G6Pase in the PKCβ^Hep–/–^ liver by insulin. FoxO1 has been reported to activate its expression in the liver; therefore, loss of FoxO1 protein in the PKCβ^Hep–/–^ liver can impair its binding to the proximal G6Pase promoter and thereby diminish its synergistic interaction with HNF-4α, resulting in lowering of G6Pase expression. It is also conceivable that PKCβ deficiency may potentiate the transcription function of HNF-4α by promoting nuclear localization (42) and/or by reducing ERK-1/2 activation (Figure 4B) (43).

A previous study has also shown that transcriptional activation of the GK promoter by HNF-4α and FoxO1 involves the coactivator protein CREB-binding protein/p300 (CBP/p300) (44). Given that PKC is reported to negatively regulate p300/CBP activity (45), it is possible that an increase in CBP activity in the absence of PKCβ enhances HNF-4α transactivation potential. In the case of FoxO1, formation of the FoxO-CBP/p300 complex has been shown to attenuate its activity due to acetylation (46). Lastly, hepatic PKCβ deficiency induces expression of SHP, which is reported to work as a repressor for both transcription factors (47). We speculate that both CBP and SHP may fine-tune differential regulation of the above genes by PKCβ.

In addition to an increase in long-term GK expression by a transcriptional mechanism, PKCβ deficiency can also exert a second level of regulation on GK activity by inducing expression of a cytosolic bifunctional protein PFK2/FBP2, an allosteric activator of GK activity (48). Such interaction activates and potentially stabilizes cytoplasmic GK (49, 50). Although we have not studied how PFK2/FBP2 is regulated by PKCβ deficiency, there is evidence to show that the transcription of this enzyme is upregulated by HIF-1 (51), which is also shown to induce GK expression through cooperative interaction with HNF-4α and CBP (44). An increase in HIF-1 transcript was observed in the PKCβ^Hep–/–^ liver (Figure 3C). Our results support that hepatic GK activity is closely and rapidly adapted by 2 regulatory mechanisms in PKCβ-deficient hepatocytes; long-term GK activity is regulated at the transcriptional level by activating PKCβ/HNF-4α and suppressing PKCβ/FoxO1 signaling axes. In the shorter term, GK activity is under the control of the binding protein PFK2/FBP2, a major allosteric activator of GK.

### The potential role of PKCβ in coordinating nutrient signaling and circadian rhythm of hepatic glycogen synthesis.

Hepatic glycogen synthesis exhibits a robust circadian rhythm that peaks at the end of the active phase in mammals (52). Yet a precise mechanistic understanding of how the circadian clock in the liver controls homeostasis is not understood. Interestingly, hepatic PKCβ deficiency is accompanied by significant increases in expression of SHP and Nr1d1 (REV-ErbA) (Figure 3C). SHP is not only a clock-controlled gene, it also interacts with Rev-ErbA to regulate clock genes (e.g., Npas2 and Bmal1) (53–56). Rev-ErbA is reported to buffer against aberrant responses to metabolic perturbation and provides protection against mistimed feeding cues (57). Understanding how the PKCβ/SHP/Rev-ErbA axis is coupled to metabolism may be critical for understanding the link between nutrient signaling and circadian rhythm and development of metabolic diseases as well as the impact of circadian disruptors.

**Figure 6:**
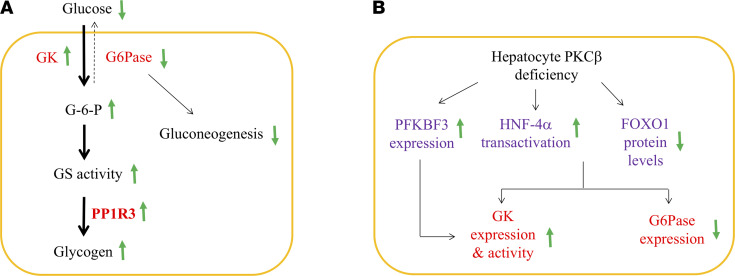
Proposed mechanisms by which hepatic PKCβ deficiency induces hepatic glycogen synthesis upon glucose overload, and the impact of PKCβ deficiency on transcription factors implicated in differential regulation of GK and G6Pase genes. Our results link PKCβ with glycogen synthesis and extend our understanding of the underlying PKCβ-dependent transcriptional mechanisms for differential regulation of hepatic GK and G6Pase genes. (**A**) PKCβ deficiency enhances conversion of glucose to glycogen after glucose exposure by coordinating induction of GK expression and suppression of G6Pase expression, in combination with simultaneous dephosphorylation/activation of GS. These changes result in elevated GK/G6Pase ratio and intracellular G6P levels and promote glycogen synthesis and storage. In line with this model, GK has been suggested to have a close functional and regulatory association with glycogen synthesis through GS. PKCβ deficiency also induces expression of GK-binding protein PFK2/FBP2, a major allosteric regulator of its activity. Since G6Pase catalyzes the last step of gluconeogenesis, PKCβ deficiency can slightly reduce gluconeogenesis at the same time. The proposed mechanism has the potential to link diet-sensitive signaling kinase PKCβ with control of glycogenesis. (**B**) A model summarizing the orchestrated role of PKCβ in the differential regulation of hepatic GK and G6Pase genes in vivo. In this model, PKCβ contributes to the regulation of these genes through suppressing HNF-4α transactivation and enhancing FoxO1 protein stability. Thus, PKCβ deficiency enhances the ability of HNF-4α to transactivate and reduces the displacement of HNF-4α by FoxO1 from GK promoter, thereby resulting in induction of the GK promoter. At the same time, FoxO1 has also been reported to activate G6Pase expression through its synergistic interaction with HNF-4α; therefore, reduction of FoxO1 can also lead to decreased expression of the G6Pase gene. Up and down arrows indicate an increase or decrease, respectively, in protein level or activity in the liver.

A model that follows from the above discussion is that the diet-sensitive PKCβ induces a coordinated response that negatively influences full response to the glucose overload at multiple levels. According to this model, hepatic PKCβ deficiency causes hypoglycemia by first promoting G6P production via GK and subsequently redirecting more G6P to glycogen by activating GS, which is not dependent on AKT and GSK-3 phosphorylation in the liver of HFHC-fed mice (Figure 6A). The coordinated induction of GK transcription and activation of GS activity by PKCβ deficiency results in greater hepatic glycogen synthesis. The loss of FoxO1 protein abundance, in combination with increased HNF-4α function by PKCβ deficiency, can account for increased GK transcription (Figure 6B). At the same time, FoxO1 is required for a full expression of G6Pase promoter, and a reduction in its protein abundance can decrease expression of G6Pase.

The mechanism by which PKCβ promotes FoxO1 stability is not clear. Several proteins are known to interact with FoxO transcription factors, regulating their intracellular localization and/or activity, and the number of newly identified regulators is rapidly increasing (58). We expect that PKCβ-mediated phosphorylation of FoxO1 might regulate its recruitment with a number of cofactors, such as NEDD E3 ligase, proteasomal activity, or 14-3-3, to participate in FoxO1 subcellular stability. It is possible that NEDD4 E3 ligase, which also has a Ca^2+^ and phospholipid-binding C2 domain similar to the PKCβ C2 domain, could mediate PKCβ effects on FoxO1 ubiquitylation (59). PKCβ may also regulate FoxO1 degradation through regulating 20S proteasome activity. A previous study has shown that PKCβ activation results in phosphorylation of the purified 20S proteasome in vitro with a reduction in its activity (60). PKCβ knockdown using siRNA also abrogated PMA-induced proteasomal dysfunction and the accumulation of damaged proteins (61). Lastly, in the cytoplasm, 14-3-3 binding attenuates FoxO dephosphorylation and degradation (62). PKCβ may phosphorylate 14-3-3 proteins and prevent its binding to target proteins, thereby contributing to nuclear retention of FoxO1 (63). Overexpression of a phospho-mimicking mutant of 14-3-3γ (S58D mutant of PKC phosphorylation site) is reported to increase nuclear staining of FoxO1, whereas overexpression of WT or a phosphorylation-deficient mutant (S58A) of 14-3-3ε did not affect the subcellular distribution of FoxO1. It is plausible that the abundance of PKCβ expression in the liver could directly or indirectly dictate FoxO1 protein levels, thus allowing fine-tuning of the pathway based on changing nutrient conditions.

In conclusion, our studies demonstrated that hepatic PKCβ is a mediator of multiple signaling cascades and integrates dietary fat and intracellular key genes of glucose homeostasis through utilizing transcriptional and posttranscriptional mechanisms. Knowledge of the PKCβ-dependent mechanisms that mediate the postprandial suppression of glycogenesis is relevant to understanding the pathophysiology of suppression of glycogen synthesis in diabetes mellitus (39, 40). In addition, the physiological connotation of PKCβ negatively regulating both glycogenesis and HNF-4α has important implications. For example, it is known that hepatocellular carcinoma (HCC) progression is associated with downregulation of HNF-4α (64) and glycogen metabolism has an important role for cancer cell survival (65). Given activation of PKCβ by cytokines, dietary fats, metabolites, and obesity, this kinase can integrate signals from different pathways and can guide the liver response to varying energy and stress conditions. Moreover, PKCβ, commonly dysregulated in many types of cancer, may have important implications in cancer development (66, 67). Further research is required to elucidate the complete mechanistic basis for the regulation of hepatic glucose homeostasis by PKCβ under different physiological conditions.

## Methods

### Generation of liver-specific PKCβ-KO mice.

PKCβ^Hep–/–^ mice were generated by crossing PKCβ^fl/fl^ mice (generated by our laboratory) on a C57BL/6J background with transgenic mice expressing Cre recombinase-expressing under the expression of the albumin-Cre transgenic mice with a C57BL6J genetic background (The Jackson Laboratory, 000664). The resultant PKCβ^Hepfl/+^ Alb-Cre+ progeny were crossed with PKCβ^fl/fl^ mice to obtain tissue-specific KO mice. Mice without the Cre transgene were used as control mice. Genotyping was confirmed for the expression of Cre transgene and PKCβ^fl/fl^ alleles prior to the onset of studies (16). The animals were housed under controlled temperature (23°C) and lighting (12-hour light/12-hour dark cycle) with free access to water and standard mouse chow diet or HFHC diet (45% kcal fat supplemented with 1% cholesterol diet; Research Diets, D04102102) (16).

### Western blotting.

The tissues were isolated from mice in the fed condition and then immediately frozen in liquid nitrogen. Tissue homogenates were prepared, and 30–50 μg of protein was resolved on 4%–12% SDS-PAGE gels and subjected to Western blotting (12). Immunoblots were performed using antibodies against the following proteins: AKT (catalog 4685), P-AKT^Thr308^ (catalog 13038), P-AKT^Ser473^ (catalog 4060), FoxO1 (catalog 2880), FoxO3a (catalog D19A7), P-FoxO1^Ser256^ (catalog 9461), P-FoxO1^Thr24^/FoxO3a^Thr32^/FoxO4^Thr28^ (catalog 2599), FoxO1 (catalog 2880), GSK3α/β (catalog 5676), P-GSK3β^Ser9^ (catalog 5558), GS (catalog 3893), P-GS^Ser641^ (catalog 94905), P-IRS-1^Ser307^ (catalog 2381), P-IRS-1^Ser612^ (catalog 3203), P-TSC2^Ser939^ (catalog 3615), TSC2 (catalog 3612), p-mTOR^Ser2449^ (catalog 5536), mTOR (catalog 2983), and P-ERK^Thr202/Tyr204^ (catalog 9101) were purchased from Cell Signaling Technology. Antibodies against HNF-4α (catalog A2085), PGC-1α (catalog A11971), GK (catalog Ab293), G6Pase (catalog A16234), GKCR (catalog A5678), and β-actin (catalog AC026) were purchased from ABclonal, and ERK (catalog 514302) was purchased from Santa Cruz Biotechnology. Goat anti-mouse and goat anti–rabbit HRP–conjugated secondary antibodies (1:3000; Bio-Rad, 1662408EDU) were used for described experiments.

### RNA isolation and RNA-Seq.

RNA was extracted from the liver using TRIzol reagents (Invitrogen). RNA-Seq was performed to quantify gene expression by Novogene. RNA-Seq analysis was performed by Novogene using RNA extracted from the liver of 12-week-old control and PKCβ^Hep–/–^ mice on an HFHC diet. Raw FASTQ files for the RNA-Seq libraries were deposited to the NCBI Sequence Read Archive (SRA) and have been assigned the bio project number PRJNA750711.

### ITTs.

ITTs were performed as described previously (16).

### PTT challenge.

For the PTT challenge, the mice were fasted for 5 hours and then administered 2 g/kg body weight of sodium pyruvate (Sigma-Aldrich) by i.p. injection. Blood glucose concentrations were measured using a One-Touch Ultra glucometer (Lifescan Inc.) at the indicated time points.

### GTTs.

The GTT was performed via an i.p. injection of glucose at 1 g/kg body weight, and blood glucose levels were measured before and 15, 30, 60, 120, and 180 minutes after the injection. Blood glucose was measured using a glucometer. Serum insulin levels were measured using an ELISA kit (UC MMPC).

### Metabolic measurements.

Blood glucose was measured using a One-Touch Ultra glucometer (Lifescan Inc.) (14). Blood was collected using a 1 mL syringe coated in 0.5 M K_2_EDTA, and serum was collected by centrifugation at 1000*g* for 20 minutes. Insulin levels were measured by ELISA. Liver G6P concentrations were measured using an enzymatic assay kit according to the manufacturer’s instructions (G6P Assay Kit MAK014; Sigma-Aldrich).

### Measurement of liver and muscle glycogen levels.

Nonfasting male mice were euthanized, and tissue was flash-frozen in liquid nitrogen. Frozen samples (~50 μg) were homogenized in 300 μL of PBS. Homogenates were quantified for protein by the bicinchoninic acid (BCA) method. Tissue sample (4 mg of total protein for liver) was used for detection of glycogen levels by colorimetric assay protocol according to the manufacturer’s instructions (Biovision). Glycogen levels in samples were determined from a standard-curve method generated by the assay.

### In vivo insulin signaling.

After an overnight fast, mice were anesthetized with 2,2,2-tribromoethanol in PBS and injected with 5 U of regular human insulin (Novolin, Novo Nordisk) via the inferior vena cava (14). Five minutes after the insulin bolus, liver tissue was removed and frozen in liquid nitrogen. Immunoblot analysis of insulin signaling molecules was performed using liver homogenates prepared in a tissue homogenization buffer and subjected to immunoblotting. All protein expression data were quantified by densitometry using ImageJ (NIH).

### Incorporation of 2-deoxy-D-[^3^H] glucose into liver glycogen.

Incorporation of glucose into glycogen was assessed as described previously (62). As a tracer, 2-deoxy-D-[^3^H] glucose (PerkinElmer) was combined with 20% (0.2 g/mL) glucose and then administered at 2 g/kg body weight at 1 mCi/mouse by i.p. injection into 3-hour fasted mice. The liver was harvested and flash-frozen in liquid nitrogen at 30 and 60 minutes after glucose and tracer injection. Plasma glucose concentrations were measured at these indicated time points. Frozen tissue samples were homogenized and radioactivity was determined.

### GS assay.

GS activity in muscle and liver lysates was measured with or without saturating G6P and 5 mmol/L UDP glucose (4).

### Liver tissue staining.

The liver sections were prepared from mice, fixed in 4% paraformaldehyde, and embedded in paraffin. To detect polysaccharide content, slices of 5–10 μM were deparaffinized, oxidized with 0.5% periodic acid for 5 minutes, stained with Schiff reagent for 15 minutes, and then counterstained with H&E for 15 minutes.

### Luciferase reporter and transfections.

Glucokinase liver-specific 5′ flanking sequences spanning bases –1,049 to +135 relative to the liver-specific mRNA start site were amplified by using the human genomic DNA (Clonetech) with primers that had HindIII and SacI restriction endonuclease digestion sites added to the 5′ and 3′ primers, respectively, to aid in directional cloning of the amplified region. Primers were used to make the reporter gene construct (5′-ggcgagctcCCACTTGCCTCAGCTTCAGGC-3′ and 5′-ggcaagcttTTTGGGAGGCAGAGA TGCTCC-3′). The 1,184 bp HindIII-SacI genomic fragment, containing the liver-specific promoter, was cloned into the luciferase reporter plasmid pGL3 Basic (Promega) to generate pGK-Luc plasmid, and the sequences were verified by sequencing. The reporter plasmid pGK HNFm-LUC, which contains mutations in the HNF-4 sites, was generated using the QuickChange mutagenesis kit (Promega) with GK Luc as a template (WT HNF-4α 5′-TGACCTTGTGACAC-3′ to HNF-4α mutant TGAatTcGTGACAC-3′). The WT HNF-4 element is underlined and mutated bases in the construct are indicated by lowercase letters. All constructs were verified by sequencing in both directions.

HepG2 cells were cultured in DMEM supplemented with 10% FBS. Transfections were performed using Fugene-6 (Roche Applied Science); 33 ng of pRSV–β-gal was included in each experiment to control for transfection efficiency. To ensure equal DNA amounts, empty plasmids were added to each transfection. Luciferase activity was normalized for β-gal activity in the same sample. Luciferase and β-gal assays were performed in triplicate.

### ChIP assays.

For ChIP, we isolated intact chromatin from primary hepatocytes (~5 × 106 cells) by using ChIP-IT Express Kit (Active Motif) following the manufacturer’s instructions; cells were sonicated and ChIP was followed by PCR using the GoTaq qPCR Master Mix (Promega). Multiple overlapping pairs of primers were designed to cover the hepatic GK promoter from –1187 to +52 from the start point. Results shown are with primer sequences as follows: 5′-GAAGGGGGCATGTGAGTG-3′ (–219/–202) and 5′-ACTGTCTGGCTGAGTGTTGC-3′ (+33/+52). qPCR was performed at 60^o^C for 30 cycles with the above primers followed by quantification. Enrichment compared with input DNA was calculated. Results were normalized to precipitation using IgG in the fed state.

### Statistics.

All results are presented as mean ± SEM. Statistical analyses were performed using GraphPad Prism 9. When only 2 groups were analyzed, statistical significance was determined using an unpaired 2-tailed Student’s *t* test. Two-way ANOVA was used to compare the effects of different diets on 2 genotypes, and when significant differences were observed, individual mean values were compared by unpaired 2-tailed Student’s *t* test as indicated. Repeated measure-based parameters were analyzed using 2-way ANOVA followed by Bonferroni’s test. *P* less than 0.05 was considered statistically significant.

### Study approval.

All mouse experiments were conducted according to protocols approved by the Ohio State University IACUC.

## Author contributions

FH and YS conducted the experiments. MCO and KDM generated floxed and hepatocyte-specific PKCβ-deficient mouse models. KDM supervised the work, wrote the manuscript, and interpreted the results.

## Supplementary Material

Supplemental data

## Version 1 10/08/2021

Electronic publication
