# Supplementary figures and images for: Role of hepatic PKCβ in nutritional regulation of hepatic glycogen synthesis
Source: JCI Insight. 2021 Oct 8;6(19):e149023. doi: 10.1172/jci.insight.149023 (PMC8525638; doi:10.1172/jci.insight.149023)

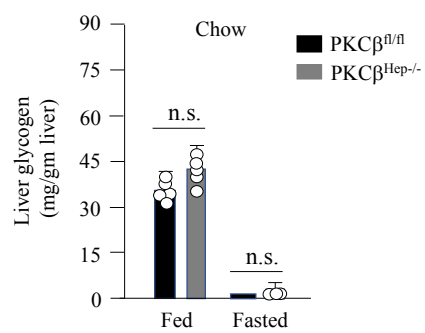

Supplementary Figure 1

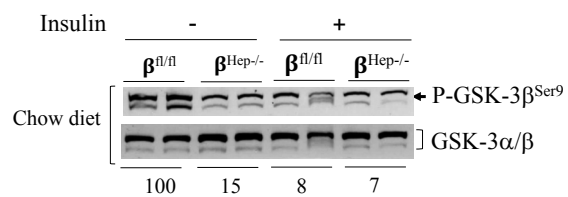

Supplementary Figure 2

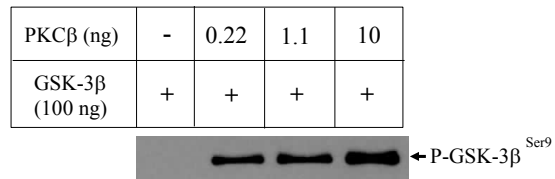

Supplementary Figure 3

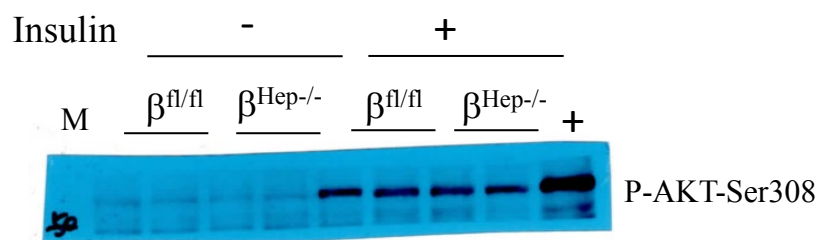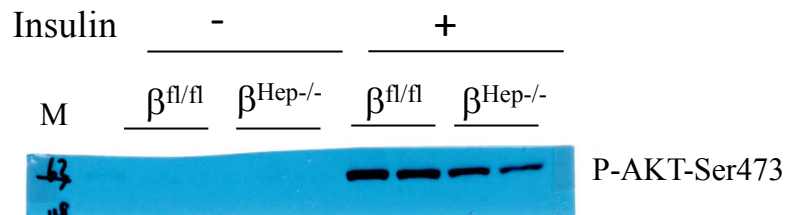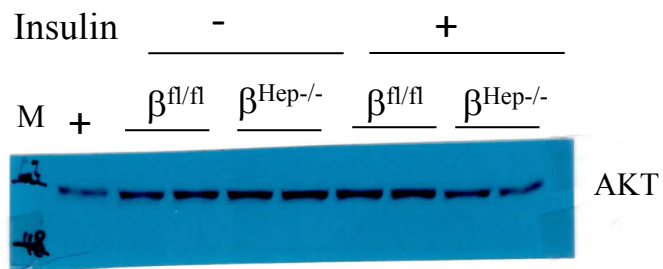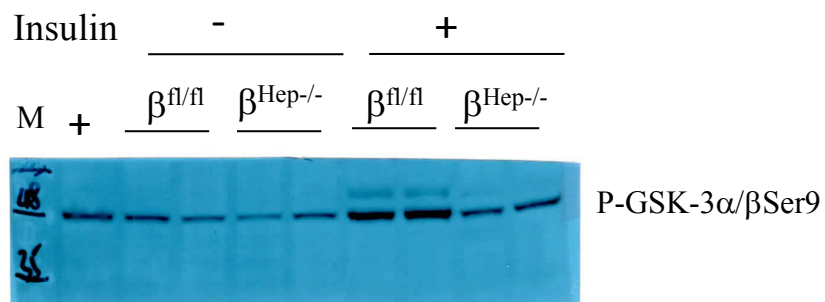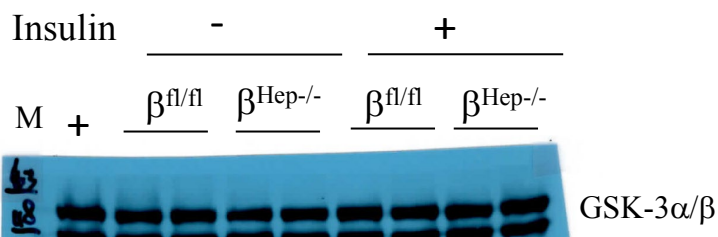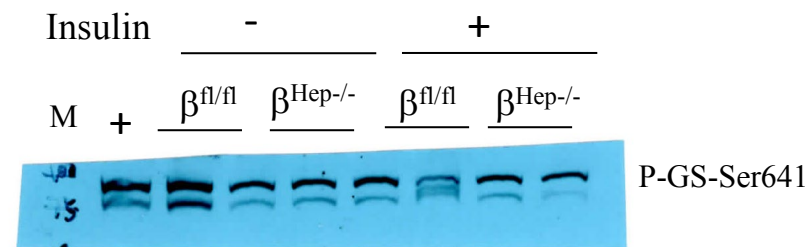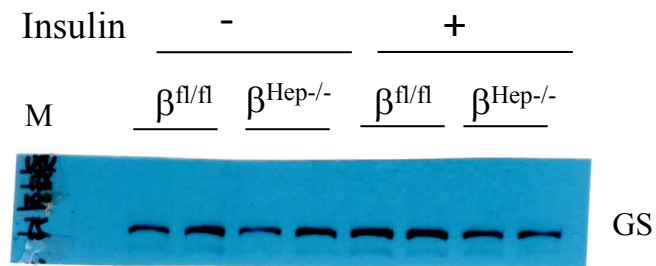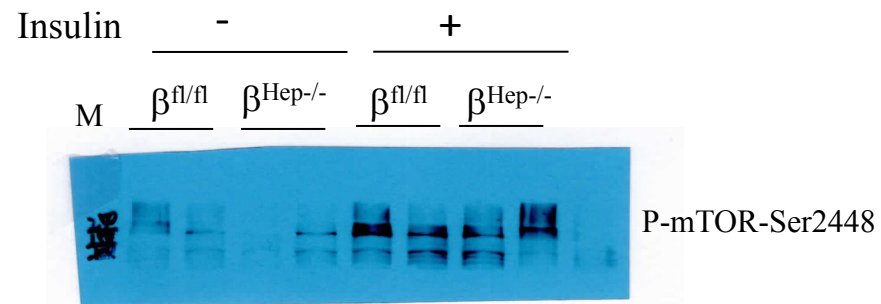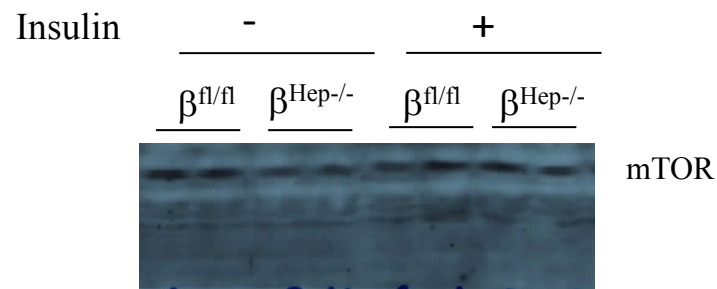

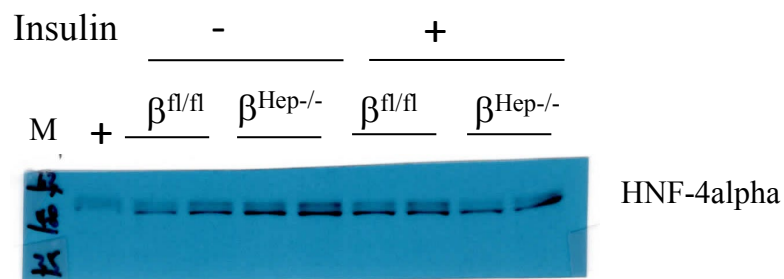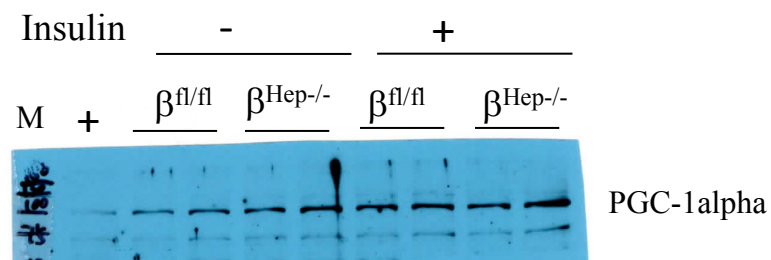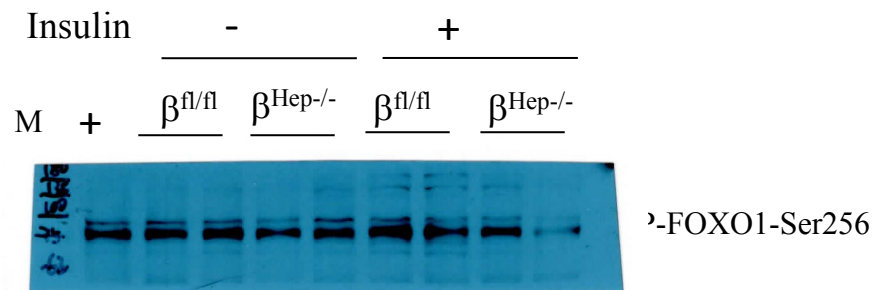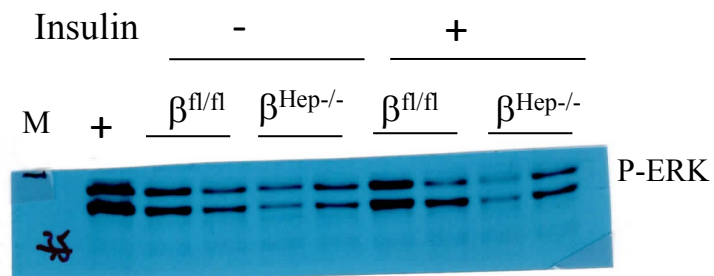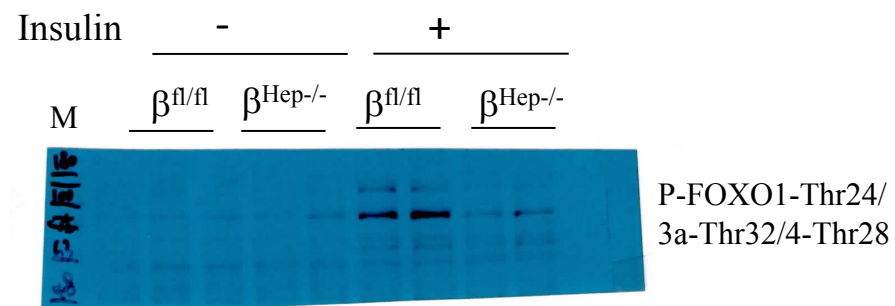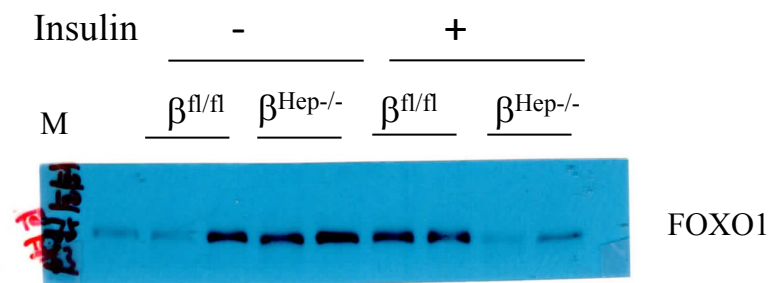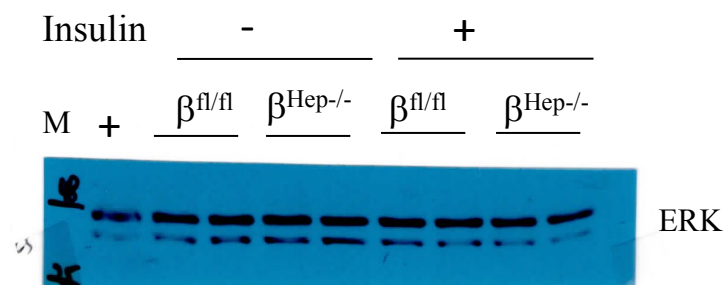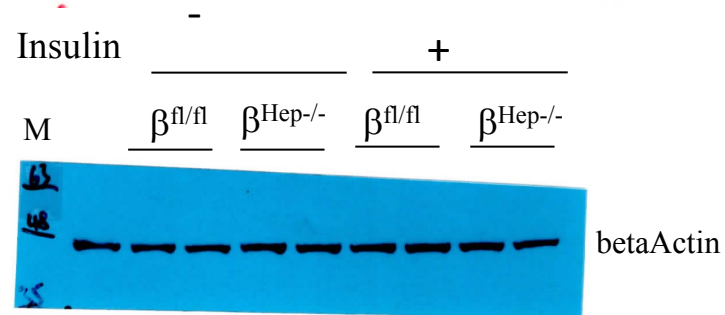

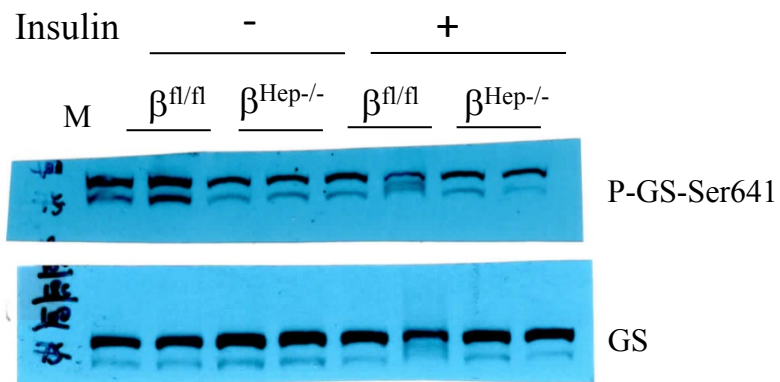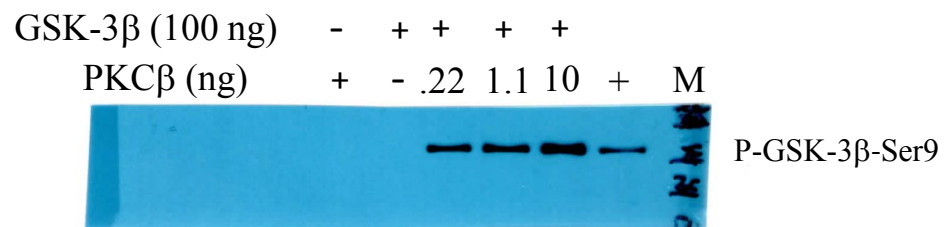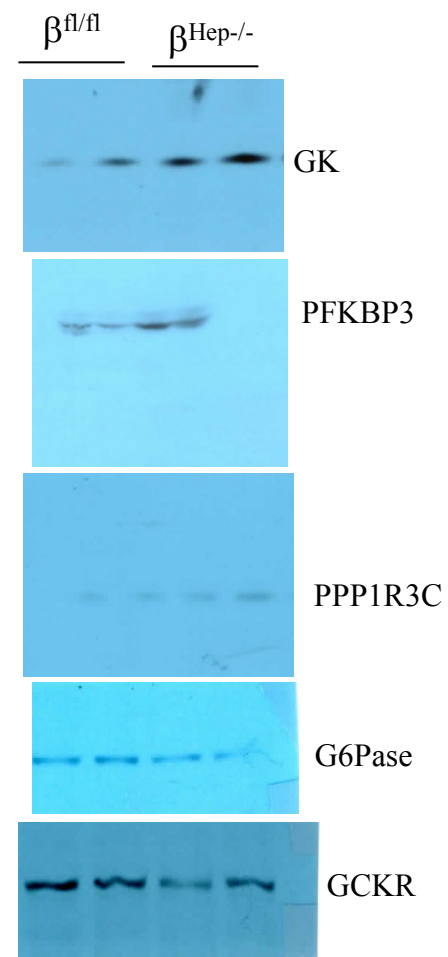

Supplement: Supplemental data [file jciinsight-6-149023-s324.pdf]
